# Adults’ Preferences for Behavior Change Techniques and Engagement Features in a Mobile App to Promote 24-Hour Movement Behaviors: Cross-Sectional Survey Study

**DOI:** 10.2196/15707

**Published:** 2019-12-20

**Authors:** Ann DeSmet, Ilse De Bourdeaudhuij, Sebastien Chastin, Geert Crombez, Ralph Maddison, Greet Cardon

**Affiliations:** 1 Clinical and Health Psychology Université Libre de Bruxelles Brussels Belgium; 2 Research Foundation – Flanders Brussels Belgium; 3 Department of Movement and Sport Sciences Ghent University Ghent Belgium; 4 Glasgow Caledonian University Glasgow United Kingdom; 5 Department of Experimental-Clinical and Health Psychology Ghent University Ghent Belgium; 6 Institute for Physical Activity and Nutrition Deakin University Geelong Australia

**Keywords:** physical activity, sleep, sedentary behavior, 24-hour movement, mobile health, mobile apps, behavior change technique, engagement, adult

## Abstract

**Background:**

There is a limited understanding of components that should be included in digital interventions for 24-hour movement behaviors (physical activity [PA], sleep, and sedentary behavior [SB]). For intervention effectiveness, user engagement is important. This can be enhanced by a user-centered design to, for example, explore and integrate user preferences for intervention techniques and features.

**Objective:**

This study aimed to examine adult users’ preferences for techniques and features in mobile apps for 24-hour movement behaviors.

**Methods:**

A total of 86 participants (mean age 37.4 years [SD 9.2]; 49/86, 57% female) completed a Web-based survey. Behavior change techniques (BCTs) were based on a validated taxonomy v2 by Abraham and Michie, and engagement features were based on a list extracted from the literature. Behavioral data were collected using Fitbit trackers. Correlations, (repeated measures) analysis of variance, and independent sample *t* tests were used to examine associations and differences between and within users by the type of health domain and users’ behavioral intention and adoption.

**Results:**

Preferences were generally the highest for information on the health consequences of movement behavior self-monitoring, behavioral feedback, insight into healthy lifestyles, and tips and instructions. Although the same ranking was found for techniques across behaviors, preferences were stronger for all but one BCT for PA in comparison to the other two health behaviors. Although techniques fit user preferences for addressing PA well, supplemental techniques may be able to address preferences for sleep and SB in a better manner. In addition to what is commonly included in apps, sleep apps should consider providing tips for sleep. SB apps may wish to include more self-regulation and goal-setting techniques. Few differences were found by users’ intentions or adoption to change a particular behavior. Apps should provide more self-monitoring (*P*=.03), information on behavior health outcome (*P*=.048), and feedback (*P*=.04) and incorporate social support (*P*=.048) to help those who are further removed from healthy sleep. A virtual coach (*P*<.001) and video modeling (*P*=.004) may provide appreciated support to those who are physically less active. PA self-monitoring appealed more to those with an intention to change PA (*P*=.03). Social comparison and support features are not high on users’ agenda and may not be needed from an engagement point of view. Engagement features may not be very relevant for user engagement but should be examined in future research with a less reflective method.

**Conclusions:**

The findings of this study provide guidance for the design of digital 24-hour movement behavior interventions. As 24-hour movement guidelines are increasingly being adopted in several countries, our study findings are timely to support the design of interventions to meet these guidelines.

## Introduction

### Movement Behaviors

Physical activity (PA), sleep, and sedentary behavior (SB) are modifiable determinants of several negative health outcomes among adults. More time spent being physically active (more light-intensity PA [LPA] and a minimum of 30-min moderate-to-vigorous PA [MVPA] per day), a sleep duration of 7-9 hours per night, and less time spent on SB are associated with beneficial health outcomes, including lower risk of weight gain and obesity [1,2], lower risk of type 2 diabetes [3-5], lower risk of cardiovascular diseases [3,6], and higher health-related quality of life [1-7]. In Europe, 61% of the adult population meets the guidelines for MVPA [8], and 73% of adults without diseases meet the recommendations for a healthy sleep duration (7-8 hours per night) [9]. Between 10% and 42% of adults in Europe have been reported sitting for more than 7.5 hours per day [10], whereas less than 7 hours of SB per day (mainly measured via self-reports) has been suggested as beneficial for health [11]. Despite the benefits of PA, reducing SB, and getting enough sleep, most studies to date have examined the duration of these movement behaviors in isolation [12]. This is problematic, as these behaviors are interrelated; over the course of 24 hours, a change in any of these given behaviors impacts the duration of (at least one) movement behavior(s). Consequently, these behaviors need to be considered together in a multibehavior program, as targeting one behavior will cause a time displacement in another behavior, and not all displacements are equally favorable to health [13,14].

### User-Centered Design of Multibehavior Programs

To date, very few multibehavior programs have been designed taking into account 24-hour movement behaviors, with no effectiveness data available thus far [15,16]. As a result, there is a limited understanding of which components should be included and how behavior recommendations are best combined in such multibehavioral interventions [17,18]. Behavior change techniques (BCTs) are uniquely identifiable components of an intervention that can be considered active ingredients of behavior change [19]. To ensure effectiveness, exposure to and active elaboration of intervention content are also required, referred to as user engagement [20]. User-centered design, in which user preferences for BCTs and other intervention features are taken into account, can help increase user engagement [20-22]. This is especially important in digital multibehavior programs that face additional challenges to user engagement because of a lack of in-person support [20]. Although BCTs are an important feature of behavior change interventions, apps may also include specific features to enhance user engagement, such as the use of celebrities [23,24], narratives [25], gamification, challenge and competition elements [25,26], interactive features such as a chat function and virtual coaches [27,28], or a social media connection [28]. In line with the Elaboration Likelihood Model, when users are not able or motivated to process the message in a rational way, the message is processed via such visual and contextual cues, here referred to as engagement features [29,30].

BCTs are best selected to fit specific determinants of behavior [31]. This may imply that different BCTs are needed and preferred by users for different behaviors. Users’ needs and preferences for techniques in an intervention may change as users proceed to adopting and maintaining the behavior change [32] and may also differ between users with a higher or lower motivation to change behavior, where those with low motivation may, for example, be more interested in peripheral cues than those with high motivation for the health behavior [32,33]. We do not expect preferences for peripheral engagement features to differ by specific behavior because these peripheral cues do not rely on content matching with specific behaviors. This is therefore not included in our study.

### Study Aims and Research Questions

To the best of our knowledge, no study has assessed adult users’ preferences for BCTs and engagement features in relation to combined PA, sleep, and SB intervention and investigated whether these differ between movement behaviors and by the user’s intention and current adoption for each health behavior domain. When designing interventions for 24-hour movement behaviors, these insights are important to ensure that users will engage with the intervention and continue to use the app for as long as it is necessary to change behavior. This study aimed to assess the following research questions: (1) What are the preferences for BCTs in mobile apps aiming to improve PA, sleep, and SB? (2) Do differences exist between participants in terms of their preferences for specific movement behavior (PA, sleep, and SB)? (3) What are their preferences for engagement features? (4) Do differences exist in participants’ preferences for BCTs or features by their intention to change the health behavior and current behavioral adoption (PA, sleep, and SB)? The results of this study may inform the evidence-based design of mobile health interventions, promoting 24-hour movement behaviors in a general adult population.

## Methods

### Study Design

The data used for this study were part of the Healthy Worker study, a 2-week intensive measurement study that assessed PA, sleep, SB, and their behavioral determinants. Incentives for study participation consisted of individual feedback and the possibility to win a folding bike via a raffle. On study completion, users completed a voluntary Web-based process evaluation survey using QuestionPro (Austin, Texas, US). The data reported here were derived from the process evaluation survey. Methods and results were reported in accordance with the Checklist for Reporting Results of Internet E-Surveys guidelines [34]. Surveys were pseudonymized to ensure confidentiality and avoid social desirability bias. The survey did not use a randomized order of items to maintain a logical flow. Adaptive questioning (branching and skips) was used to reduce respondent burden. Surveys were sent to individual email addresses, disabling multiple logins. Only completed surveys were included. Most items required a response to continue. Participants could save responses and continue later. It took, on average, 12 min to complete the survey, which consisted of 40 questions. Data were collected between February 2017 and May 2017. Participants received a personal feedback report at study completion and, as an incentive, they could win a folding bike via a raffle. The ethics committee of Ghent University Hospital approved this study (reference 2016/1231).

### Participants and Sampling Procedure

A random sample of working-age adults (aged 22-55 years) was drawn from the civil registry in Ghent, a city of roughly 250,000 inhabitants in Flanders, Belgium. A total of 2453 letters were sent out, of which 47 were returned undeliverable. Participants were included if they were aged 22-55 years and owned a smartphone with access to the internet. Participants were requested to notify the researchers of their interest to participate by email and provide their phone number and postal address. Home visits were made to each participant to set up the Fitbit app (San Francisco, California, US) and wearable device and to retrieve the Fitbit app after study completion.

### Measurements

#### Sociodemographic Information

Sociodemographic information including gender (male/or female), age (continuous), and highest educational degree (primary school, secondary school, postsecondary nonacademic education, or academic education) was assessed in a Web-based survey 1 week before the start of the study.

#### Movement Behaviors

Participants were provided a Fitbit Charge 2, a commercial wrist-worn activity tracker that includes a triaxial accelerometer and a heart rate monitor. Fitbit devices have been shown to be valid and reliable commercial devices to measure the time spent on these movement behaviors [35,36]. The Fitbit Charge 2 measured minute-by-minute activities classified as LPA, moderate PA (MPA), vigorous PA (VPA), sleep, or SB. The usability of the Fitbit trackers was assessed with Likert scale items (1-5) based on the Short Usability Scale [37] and was rated as high, with average scores between agree and completely agree (≥4 or 5) for ease of use, self-confidence in using it, quickly learning how to use it, good integration of functionality, and interest in using it in the future. Full descriptive results are provided in Multimedia Appendix 1.

#### Preferences for Behavior Change Techniques and Engagement Features in a Mobile App on Physical Activity, Sedentary Behavior, and Sleep

Preferences for BCTs and engagement features were rated per behavior on a 5-point rating scale. A short BCT list, consisting of 22 BCTs (Multimedia Appendix 2) [38] rather than the more extensive list of 93 BCTs [19], was used to reduce respondent burden. The BCT list was inspired by commonly used techniques in health apps [28,39,40] when scored based on BCT taxonomy of Abraham and Michie [38]. Participants rated their preferences for specific engagement app features on a 5-point rating scale including social media connection [28], gamification [25,26], competition with others [25], a virtual coach to give instructions [27,28], being able to ask questions via a chat function [27], having a narrative (eg, fictional drama) and being a character in this narrative [25], and having celebrity endorsement of the app [24].

#### Intention to Change Movement Behaviors

Validated scales to measure intention-to-change behavior were used for each behavior, where possible. As a result, the operational definitions of intentional phases differed between each health behavior. Intention was measured before the start of the study in a Web-based survey for PA and SB and in diaries during the 14-day follow-up period for sleep. For PA, the intention phase was determined by one item from the Belgian Environmental Physical Activity Study survey reflecting their stages of change: (1) premotivational phase: “I am not sufficiently active and have no intention to change in the next 6 months,” (2) action phase: “I am not sufficiently active and have the intention to change in the next 6 months,” and (3) maintenance phase: “I am sufficiently physically active” [41]. The action and maintenance phases were grouped into the intentional phase. For sleep, no validated behavioral determinant questionnaires existed then. A questionnaire was developed in the framework of this study and validated elsewhere (DeSmet et al, unpublished, 2019). The intention to improve sleep was defined as users’ intention to go to bed on time (measured in evening diaries, averaged over 14 days, and dichotomized by no intention ≤3 [*completely disagree to neutral*] and >3 [*rather to completely agree*] on a 1- to 5-point rating scale). For SB, users were asked for their intention to reduce their time spent sitting based on a previous scale for occupational sitting time [42], assessed here across five different domains (ie, while watching TV, using computers in leisure time, during other leisure activities, during transport, and at work). If users indicated they wanted to reduce their sitting in at least one of these areas in the next 6 months, they were considered to have an intention to reduce their SB.

### Analysis

Descriptive statistics assessed participants’ average preferences for BCT by each movement behavior and for app features (research question 1). Associations of preferences with age were assessed with Pearson correlations, and differences in preferences by gender were assessed with analysis of variance (ANOVA) tests. Repeated measures ANOVA analyses were conducted to assess differences among users in their preferences for BCTs in each of the three movement behaviors (PA, sleep, and SB). No between-factor test was used. Significant results were subsequently tested via independent sample *t* tests to detect differences between pairs (research question 2). ANOVA tests were used to assess differences among participants by their intention to change and current behavior in relation to PA, sleep, and SB. Homogeneity of variances was assessed, and significance tests were only performed for item sets with nonsignificant tests on homogeneity of variances (research question 3). All statistics were performed in SPSS, version 25 (IBM corporation, Armonk, NY).

## Results

**Figure 1:**
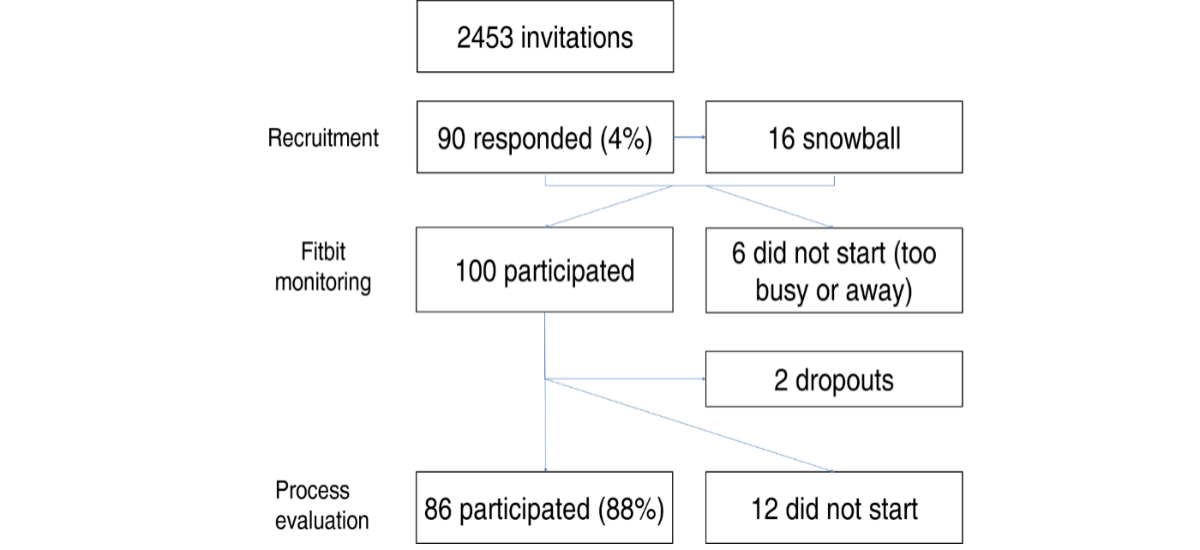
Study flow.

A total of 98 participants completed the main study, with 86 participants completing the process evaluation (response rate 88%; Figure 1). The analyzed sample (n=86) mostly consisted of people living in an urban area (65/86, 76%), who were well educated (71/86, 83% completed postsecondary education), living together with a partner (58/86, 67%), and working full time (66/86, 77%). The mean age was 37.4 years (SD 9.2), and 57% (49/86) of the participants were female. Fitbit data were, on average, available for 11.9 of 14 days (median 14, SD 4.3). Participants spent, on average, 4.6 hours (SD 1.19) on LPA, 25.3 min (SD 15.01) on MPA, 29.4 min (SD 20.7) on VPA, 7.6 hours (SD 0.80) asleep, and 10.3 hours (SD 1.31) on SB per 24 hours. There were no significant correlations between the number of days Fitbit data were available for and users’ activity profile. Of the 74 preference items, there were significant correlations with one technique and one feature (ie, having more measured days was positively correlated with a preference for social comparison with others on PA: r=0.22, *P*=.04; having more measured days was negatively correlated with a preference for a narrative: r=−0.22, *P*=.04).

Across movement behaviors, the same BCTs were the most preferred, that is, information on the health consequences of movement behaviors, self-monitoring of behavior, feedback on how well they do, obtaining insight into their healthy lifestyles, and receiving tips and instructions on how to do the behavior (Multimedia Appendix 2). Moreover, the standard deviations for preferences on the specific techniques of *information on the link between behavior health outcome* and *behavioral self-monitoring* were small, indicating a strong agreement among users for these BCTs. Although the same BCTs were ranked highest across behaviors, there were significant differences in participants’ preferences for the BCT items between the health domains of PA, sleep, and SB on all but one BCT (ie, time management skills; Multimedia Appendix 2). There was a higher preference for several BCTs to promote PA than for sleep or SB. This was the case for several BCTs associated with goal setting (ie, setting and adjusting personally desired outcomes, setting and adjusting personally relevant goals, building up toward more difficult goals, and receiving a reminder). This was also the case for several BCTs in relation to positive reinforcement (ie, receiving rewards of incentives, providing encouragement, and obtaining social support) and BCTs related to (role) modeling (ie, receiving videos that model the desired behavior and being a role model for others).

Specific BCTs were less preferred for SB than for sleep and PA. This was noted for several BCTs in relation to instructions to perform the behavior, self-monitoring, and feedback (ie, information on link between behavior and health outcome, instructions on how to perform the behavior, self-monitoring of behavior, feedback on behavior, and receiving regular feedback on how the behavior contributes to the health outcome). Identifying barriers for the behavior was less preferred for SB than for sleep and PA. For two BCTs— receiving tips tailored to their profile and to compare themselves with others with a similar profile—there was a lower preference for SB than for PA, but there was no difference with sleep. For obtaining insights into the differences between what the person does and what is needed to meet the health outcome, the preference was significantly higher for PA than for the other behaviors, and this BCT preference for sleep was significantly higher than for SB. In sum, the preference was lowest for SB on all BCTs but one (no significant difference in time management skills). Goal setting, role modeling, and reinforcement BCTs were generally preferred more for PA than for either sleep or SB. Instructions, behavioral monitoring, and feedback were less preferred for SB than for either sleep or PA.

The preference for engagement features was low to very low (Multimedia Appendix 3).

Very few BCTs showed a difference in preference between participants with a low or high intention to change the specific movement behavior (Multimedia Appendices 4-6.) For PA only, participants with a higher intention to increase this behavior had a stronger preference for monitoring their behavior than participants with a lower intention. There were no significant differences by intention to change behavior on any other BCT preference (Multimedia Appendices 4-6). Participants who had fewer hours of sleep per night showed a higher preference for information on behavior health outcome, behavioral monitoring, and feedback (Multimedia Appendix 5). For PA, lower levels of MVPA were associated with a higher preference for a virtual coach that gives instructions and for video modeling (Multimedia Appendix 4).

## Discussion

### Principal Findings

When designing interventions for 24-hour movement behaviors, it is important to assess whether users would prefer a different approach for each behavior. This study examined adult users’ preferences for different BCTs in a mobile app to improve 24-hour movement behaviors of PA, sleep, and SB and to assess whether these preferences differed by behavior and users’ behavioral intention and adoption. The study also examined preferences for engagement features and differences in preferences by users’ behavioral intention and adoption.

In general, the same BCTs were top-ranked across 24-hour movement behaviors, although the strength of these preferences was higher for PA than for SB or sleep. This suggests that the same BCTs can be used universally for combined intervention on the 24-hour movement behavior rather than behavior-specific BCTs. For all behaviors, information on behavior health outcomes, obtaining insights into their healthy lifestyles, self-monitoring, feedback, instructions, or tips were ranked the highest, and social support and comparison were ranked lowest in user preferences. The high preference for self-monitoring techniques is in line with earlier research on user preferences for apps to promote PA [28] and underscores the upcoming interest among users in commercial wearable activity trackers that monitor PA, sleep, and SB. In Belgium, 19% of adults had a wearable activity tracker in 2017 compared with only 5% owning one in 2016 [43]. Some BCTs, such as self-regulation techniques, were less preferred for both sleep and SB than for PA. A lower preference for self-regulation techniques, such as action and coping planning, to improve sleep in comparison with PA, aligns with previous research showing that users felt that self-regulation techniques were not always easy to apply, given limited control over wake time and bedtimes [44], or where integrating self-regulation techniques in mental imagery was found more useful to promote sleep than the traditional form of creating implementation intentions, given the habitual nature of bedtime routines [45].

This study moreover examined preferences and differences in user preferences for BCTs and engagement features by behavioral intention and adoption. The interest in the engagement features was generally low. A previous study on engagement features in digital health interventions to reduce alcohol consumption showed that personalization, control features (being able to make choices), and interactive features (allowing you to enter information and take a game or quiz) were most appreciated. Action plans and challenge features (competition against others) were ranked as least important by some, but as more important by others in the study. Narrative features (storyline, in which the user can be a character) were rated as not very important for engagement with the app [25]. This largely fits with our findings. This earlier study found little consistency in user preferences [25]. For example, it showed that users only valued social comparison features if they expected that they would not be outperformed by others; however, our study did not find a higher user preference for such features among users with a behavior that was in a healthier range compared with those who scored worse on these behaviors. We also found no difference in preferences for engagement features by intentional phase, disconfirming our expectation that these features would appeal more via a peripheral route among those with a lower motivation. However, our self-reported method of assessment may not have been appropriate to capture any interest in features via a peripheral route. For example, in entertainment education, where a narrative and characters are used in an entertainment format to convey health messages, a crucial condition for its effectiveness is that the message is provided unobtrusively and that the audience is unaware of the intent to change their opinions or behavior [46]. An experimental method or a conjoint analysis method where examples of apps differing in these features are shown and rated by users, rather than asking them to reflect on the importance of these features, may be better suited to assess user preferences for such engagement features in apps to promote PA, sleep, and SB.

A stronger preference for BCTs of self-monitoring, reminders or cues, role modeling, and rewards for PA than for SB and sleep may be unexpected, as these BCTs are mainly assumed to change automatic, habitual behavior [31,47,48] and would hence be expected as more preferred for SB and sleep than for PA. Possibly, a higher intention to change PA and familiarity with PA may have resulted in a stronger preference for BCTs to change this movement behavior than for SB or sleep. This is, however, not supported by our findings for SB, as no difference was noted in BCT preferences between participants with an intention to change SB and those without such an intention nor were there any significant associations found between the time spent on SB and any of the BCT preferences. For sleep, however, those with a longer sleep duration were indeed less interested in using self-monitoring for sleep. The lower preferences for SB and sleep on these BCTs than for PA may also be a consequence of the taxonomy that was used. A relatively brief set of BCTs was selected to reduce respondent burden when scoring these for three behaviors. The taxonomy used was inspired by previous scorings of mobile health apps [28,39,40] but was designed specifically for PA and diet interventions [38,49]. Possibly for sleep and SB, other relevant techniques may need to be included, such as sleep hygiene practices and cognitive behavioral therapy for sleep, or environmental changes, emotional persuasion, and education for SB [50-52], which were not examined here. A selection of BCTs that is relevant for SB and sleep, for example, based on a larger taxonomy of 93 BCTs has been constructed with expert input from several behavioral areas [19], may be appropriate for future studies on user ratings of techniques to address 24-hour movement behaviors. Finally, we may expect that features differentially implemented across behaviors in the Fitbit that the participants had worn before filling out this survey may have affected their preference ratings. This does not appear to be the case, as preferences were higher for PA both for BCTs more strongly integrated for PA than for other behaviors (eg, detailed and real-time self-monitoring), as well as for features more strongly implemented for SB than for PA (ie, reminders and buzzes).

Some recommendations can be made when comparing user preferences with what is currently commonly used in apps. PA tracker apps often include information, self-monitoring, instructions and feedback, goal-setting techniques, social support, modeling, and social comparison [53]. Our findings show that all BCTs, except social comparison techniques, that showed the lowest preference are important to include in PA apps to ensure user engagement. Sleep tracker apps include fewer BCTs and mainly consist of self-monitoring and feedback [53], which align well with preferred BCT factors for sleep observed in our study. Our findings suggest that adding *giving tips* as BCT in sleep apps may further increase user engagement. In addition, for SB, activity tracker apps mostly include self-monitoring and feedback [53]. A previous study suggested including more social support features in SB apps [53]. On the basis of our findings, we expect that this would not increase user engagement, as preferences were lowest for social comparison and support. Self-regulation and goal-setting techniques, however, were highly preferred and may be a more useful addition to SB apps. In addition, two mobile apps have been designed to improve multibehavior patterns of PA, sleep, and SB in adults: *Balanced* and *BeWell24*. *Balanced* included goal setting, information on behavior health outcome, self-monitoring, and feedback as BCTs [16]. *BeWell24* included goals and planning, feedback, and monitoring, shaping knowledge, and associations for all behaviors, complemented with shaping knowledge, natural consequences, and repetition and substitution for some behaviors [15]. It appears that these fit well with user preferences for these behaviors, with the exception of goal setting, which is included but not highly preferred for all behaviors and tips and instructions, which are highly preferred but not included.

In addition, for BCTs, we did not find any differences according to users’ intentions to improve their behaviors. Those with a lower intention to change their behavior may be more interested in BCTs that increase risk perception and positive outcome expectancies, such as feedback and information on the behavior health link [31], and those with a higher intention may have a higher preference for self-regulation techniques [32]. This was not consistent with our results; however, we did observe a higher interest in these features, not by intention, but by behavioral adoption of sleep: Participants who had fewer hours of sleep per night showed a higher preference for information on behavior health outcome, behavioral monitoring, and feedback. Thus, these techniques may be important to create awareness and initiation of health behavior change [54]. Other specific BCTs and engagement features also appeared to be preferred by people who showed lower levels of healthy behavior, indicating that these may be especially useful to support initial behavior change. Those with already higher levels of sleep wished to receive more social support for their sleep. Those with a high intention to change PA were more interested in self-monitoring their behavior. For PA, lower levels of MVPA were associated with a higher preference for a virtual coach that gives instructions and for video modeling. This suggests that more instructions and support are needed on specific exercises via which to actually achieve more MVPA. Such virtual coaches have been integrated into several research-grade mobile apps or digital interventions for PA [55-57], but in commercial settings, apps are often provided at a premium (eg, *Fitbit Coach*), although some trackers have also started providing free audio guidance during sports activities (eg, *Polar Beat* and Moov). From a public health perspective, it is unfortunate that a virtual coach would be offered at a premium because it would then be available only to those who can afford it. On the other hand, this is where an opportunity may lie for public health and academic organizations to develop such an app that incorporates a virtual coach and can be provided free as an add on to the service provided by commercial apps and wearable trackers.

### Limitations and Strengths

The study had some limitations. Despite using a random sampling method to recruit a sample representative of the population, the final sample was highly educated. This is a common problem in health promotion research. The Belgian National Health Survey, a nationally representative survey conducted among 12,038 Belgian individuals aged 15 years or older, with 3191 individuals from the Flemish region, shows that 48% of Flemish individuals have attained postsecondary education compared with 83% in our sample of individuals aged 22-55 years [58]. Although some differences may result from the inclusion of a younger age group that could not have attained postsecondary education yet (ie, aged 15-21 years, approximately 9%), it is clear that our sample overrepresents people who are highly educated. This may have had an influence on behavior outcomes, as both SB and PA were higher among well-educated individuals than among less-educated people in the Belgian National Health Survey [59]. It is unclear how this may have affected the preferences for BCTs, as we are not aware of any studies assessing the differences in preferences for BCTs by educational background. We could, however, expect a lower preference for self-quantification among less educated individuals based on existing research [60]. Our findings may thus not be generalizable to users who are less educated. Our list of chosen BCTs was based on previous research on mobile health apps. A more extensive list of BCTs may have uncovered more unmet needs of users in current mobile apps. Preferences were elicited via self-report measures in a hypothetical way. Future research should validate these findings by using experimental studies with experiences from an actual app. Eliciting user preferences is the first step in a user-centered design of a digital health intervention. On the basis of these user preferences, a prototype can be developed that can be optimized based on subsequent rounds of user feedback [61].

This study also had several strengths. It examined BCT preferences for each behavior separately, indicating important differences that should be taken into account in 24-hour activity interventions. The study examined not only BCTs but also other features that may increase engagement. As engagement and effectiveness are closely entwined, examining engagement features is important in intervention design considerations. Finally, examining differences between users in their preferences provides a useful basis for tailoring 24-hour activity interventions.

### Conclusions

Across behaviors, preferences were generally highest for information on the link between behavior health outcomes, self-monitoring, behavioral feedback, insight into healthy lifestyles, and tips and instructions. In general, very few differences were found by users’ intentions or adoption to change a particular movement behavior. The same ranking of preferred BCTs was found for all 24-hour movement behaviors, indicating that the same selection for all three behaviors could fit user preferences. However, some techniques may be added to more closely meet preferences for sleep and SB. In addition to what is commonly included in apps, sleep apps should consider giving tips for sleep and provide more information on behavior health outcome and feedback to support those who are further removed from healthy sleep. A virtual coach and video modeling may provide appreciated support to those who are less physically active. Social comparison and support features are not high on users’ agenda and may not be needed from an engagement point of view. Engagement features may not be very relevant for user preferences but should be examined in future research with a less reflective method.

## Appendix

Multimedia Appendix 1 Perceived usability of the Fitbit tracker.

Multimedia Appendix 2 Repeated measured analyses assessing within-user differences in behavior change technique preferences between health domains.

Multimedia Appendix 3 Descriptive statistics for engagement features.

Multimedia Appendix 4 Differences between participants in behavior change technique preferences for physical activity by users’ intention to change behavior and behavioral adoption.

Multimedia Appendix 5 Differences between participants in behavior change technique preferences for sleep by users’ intention to change behavior and behavioral adoption.

Multimedia Appendix 6 Differences between participants in behavior change technique preferences for sedentary behavior by users’ intention to change behavior and behavioral adoption.

## Abbreviations

BCT: behavior change technique
LPA: light physical activity
MPA: moderate physical activity
MVPA: moderate-to-vigorous physical activity
PA: physical activity
SB: sedentary behavior
