# Supplementary material for: Adults’ Preferences for Behavior Change Techniques and Engagement Features in a Mobile App to Promote 24-Hour Movement Behaviors: Cross-Sectional Survey Study
Source: JMIR Mhealth Uhealth. 2019 Dec 20;7(12):e15707. doi: 10.2196/15707 (PMC6942183; doi:10.2196/15707)
Supplement: Multimedia Appendix 1 [file mhealth_v7i12e15707_app1.docx]

**Supplementary material**

*Table Appendix 1. Perceived usability of the FITBIT tracker*

|  | **M ± SD** |
| --- | --- |
| The Fitbit was easy to use | 4.4 ± 0.8 |
| I felt self-confident in using the Fitbit | 4.3 ± 0.7 |
| Most people would quickly learn how to use the Fitbit | 4.2 ± 0.6 |
| Several possibilities and functions were well integrated in the Fitbit | 4.1 ± 0.7 |
| I would like to use the Fitbit more often | 4.0 ± 1.2 |
| I doubted the accuracy of the Fitbit data on my sleep | 2.9 ± 1.2 |
| I doubted the accuracy of the Fitbit data on my physical activity or sitting time | 2.7 ± 1.2 |
| There were too many inconsistencies in the product | 1.9 ± 0.8 |
| The Fitbit disturbed me during my sleep | 1.9 ± 1.1 |
| The Fitbit was uncomfortable to use | 1.8 ± 0.9 |
| I had to learn several things before I could get started with the Fitbit | 1.7 ± 0.8 |
| The Fitbit disturbed me during sport- or movement activities | 1.7 ± 0.8 |
| The Fitbit was too complicated | 1.5 ± 0.5 |
| I needed the help of a technical person to use the Fitbit | 1.3 ± 0.5 |

|  |
| --- |
