# Supplementary material for: Adults’ Preferences for Behavior Change Techniques and Engagement Features in a Mobile App to Promote 24-Hour Movement Behaviors: Cross-Sectional Survey Study
Source: JMIR Mhealth Uhealth. 2019 Dec 20;7(12):e15707. doi: 10.2196/15707 (PMC6942183; doi:10.2196/15707)
Supplement: Multimedia Appendix 2 [file mhealth_v7i12e15707_app2.docx]

**Multimedia Appendix 2.** Repeated measured analyses assessing within-user differences in BCT preferences between health domains

| **Behavior change techniques**  (in decreasing order by preferences for PA)  **n=85** | **Physical activity** (b)  **%Top2 box M±SD** | **Sleep** (a)  **%Top2 box M±SD** | **Sedentary behavior** (c)  **%Top2 box M±SD** | **F**  ***P*** |
| --- | --- | --- | --- | --- |
| BCT1: Info behavior-health outcome | 97.6% | 94.1% | 83.5% | **9.87** |
|  | M=4.52±0.59 ^c^ | M=4.46±0.68 ^c^ | M=4.19±0.84 ^a, b^ | ***<.001*** |
| BCT2: Self-monitoring of behavior | 96.5% | 91.8% | 83.5% | **8.06** |
|  | M=4.56±0.61 ^c^ | M=4.49±0.68 ^c^ | M=4.22±0.88 ^a, b^ | ***.001*** |
| BCT3: Feedback on how well I do with [sleep, PA, SB] | 91.8% | 88.2% | 76.5% | **9.23** |
|  | M=4.35±0.74 ^c^ | M=4.25±0.72 ^c^ | M=3.95±0.99 ^a, b^ | ***<.001*** |
| BCT11: Getting insight in difference between what I do and what is needed to achieve the desired outcome | 90.6% | 88.2% | 80.0% | **8.17** |
|  | M=4.33±0.75 ^a, c^ | M=4.21±0.77 ^b, c^ | M=4.01±0.92 ^a, b^ | ***.001*** |
| BCT18: Getting tips tailored to my profile in relation to [sleep, PA, SB] | 84.7% | 84.7% | 78.8% | **4.40** |
|  | M=4.12±0.78 ^c^ | M=4.05±0.77 | M=3.92±0.90 ^b^ | ***.02*** |
| BCT10: Regular feedback on how [sleep, PA, SB] contributes to desired outcome | 83.5% | 82.4% | 76.5% | **5.31** |
|  | M=4.14±0.88 ^c^ | M=4.08±0.82 ^c^ | M=3.93±0.94 ^a, b^ | ***.01*** |
| BCT4: Instructions how to improve [sleep, PA, SB] | 83.5% | 81.2% | 69.4% | **5.95** |
|  | M=4.20±0.84 ^c^ | M=4.14±0.90 ^c^ | M=3.89±1.02 ^a, b^ | ***.004*** |
| BCT6: Adjusting personal goals | 77.6% | 63.5% | 63.5% | **9.31** |
|  | M=4.00±1.00 ^a, c^ | M=3.78±0.99 ^b^ | M=3.68±1.07 ^b^ | ***<.001*** |
| BCT5: Setting personal goals | 76.5% | 62.4% | 62.4% | **8.79** |
|  | M=4.06±0.98 ^a, c^ | M=3.78±0.97 ^b^ | M=3.66±1.08 ^b^ | ***<.001*** |
| BCT8: Setting a personally desired outcome | 74.1% | 64.7% | 60.0% | **11.79** |
|  | M=4.05±1.05 ^a, c^ | M=3.82±1.08 ^b^ | M=3.69±1.09 ^b^ | ***<.001*** |
| BCT13: Identifying barriers for [sleep, PA, SB] | 72.9% | 76.5% | 63.5% | **4.18** |
|  | M=3.91±0.98 ^c^ | M=3.89±0.94 ^c^ | M=3.71±0.96 ^a, b^ | ***.02*** |
| BCT9: Adjusting my personally desired outcome | 70.6% | 60.0% | 60.0% | **8.85** |
|  | M=3.89±1.02 ^a, c^ | M=3.66±0.98 ^b^ | M=3.61±.04 ^b^ | ***<.001*** |
| BCT7: Gradually building up to more difficult goals | 69.4% | 55.3% | 52.9% | **10.49**  ***<.001*** |
|  | M=3.84±1.08 ^a, c^ | M=3.54±0.98 ^b^ | M=3.51±1.09 ^b^ |  |
| BCT17: Getting time management tips that help me improve my [sleep, PA, SB] | 69.4% | 69.4% | 63.5% | 2.78 |
|  | M=3.74±1.03 | M=3.68±1.01 | M=3.55±1.08 | *0.07* |
| BCT20: Getting a reminder when it is time to do something about my [sleep, PA, SB] | 67.1% | 55.3% | 55.3% | **9.78** |
|  | M=3.66±1.13 ^a, c^ | M=3.38±1.15 ^b^ | M=3.35±1.24 ^b^ | ***<.001*** |
| BCT21: That the app provides encouragement and helps to keep it up | 63.5% | 47.1% | 49.4% | **10.94** |
|  | M=3.73±1.05 ^a, c^ | M=3.36±0.96 ^b^ | M=3.39±1.15 ^b^ | ***<.001*** |
| BCT12: Creating an action plan for [sleep, PA, SB] | 60.0% | 49.4% | 50.6% | **6.87** |
|  | M=3.76±1.09 ^a, c^ | M=3.53±1.02 ^b^ | M=3.48±1.08 ^b^ | ***.002*** |
| BCT15: To compare myself with others with a similar profile of [sleep, PA, SB] | 54.1% | 51.8% | 44.7% | **4.07** |
|  | M=3.27±1.23 ^c^ | M=3.19±1.23 | M=3.08±1.21 ^b^ | ***.02*** |
| BCT22: Getting a reward, incentive or appreciation when I make progress in relation to [sleep, PA, SB] | 47.1% | 37.6% | 37.6% | **7.98** |
|  | M=3.27±1.21 ^a, c^ | M=3.08±1.09 ^b^ | M=3.02±1.23 ^b^ | ***.001*** |
| BCT19: Getting video’s that show me how to improve my [sleep, PA, SB] | 43.5% | 36.5% | 38.8% | **6.32** |
|  | M=3.15±1.18 ^a, c^ | M=2.93±1.25 ^b^ | M=2.96±1.24 ^b^ | ***.003*** |
| BCT14: Getting social support to improve my [sleep, PA, SB] | 41.2% | 27.1% | 31.8% | **8.70** |
|  | M=3.07±1.13 ^a, c^ | M=2.75±1.17 ^b^ | M=2.82±1.15 ^b^ | ***<.001*** |
| BCT16: That I can be an example to others, inspire or motivate them for [sleep, PA, SB] | 25.9% | 22.4% | 22.4% | **3.85** |
|  | M=2.74±1.19 ^a, c^ | M=2.64±1.10 ^b^ | M=2.62±1.10 ^b^ | ***.03*** |

%Top2 box refers to % of respondents answering 4 or 5 on a 5-pt rating scale
