# Supplementary material for: Adults’ Preferences for Behavior Change Techniques and Engagement Features in a Mobile App to Promote 24-Hour Movement Behaviors: Cross-Sectional Survey Study
Source: JMIR Mhealth Uhealth. 2019 Dec 20;7(12):e15707. doi: 10.2196/15707 (PMC6942183; doi:10.2196/15707)
Supplement: Multimedia Appendix 3 [file mhealth_v7i12e15707_app3.docx]

**Multimedia Appendix 3.** Descriptive statistics for engagement features

| **Engagement features** (in decreasing order of user preference) | **%Top2 box, M ± SD** |
| --- | --- |
| Virtual coach | 53.6%, M=3.17 ± 1.24 |
| Competition with others | 28.6%, M=2.57 ± 1.19 |
| Chat function | 26.2%, M=2.51 ± 1.14 |
| Gamification | 17.9%, M=2.55 ± 1.00 |
| Narrative | 4.8%, M=1.67 ± 0.86 |
| Being a character in the narrative | 3.6%, M=1.67 ± 0.86 |
| Endorsed by celebrities | 2.4%, M=1.45 ± 0.68 |
| Connection to social media | 1.2%, M=1.67 ± 0.77 |

%Top2 box refers to % of respondents answering 4 or 5 on a 5-pt rating scal
