# Supplementary material for: Adults’ Preferences for Behavior Change Techniques and Engagement Features in a Mobile App to Promote 24-Hour Movement Behaviors: Cross-Sectional Survey Study
Source: JMIR Mhealth Uhealth. 2019 Dec 20;7(12):e15707. doi: 10.2196/15707 (PMC6942183; doi:10.2196/15707)
Supplement: Multimedia Appendix 4 [file mhealth_v7i12e15707_app4.docx]

**Multimedia Appendix 4.** Differences between participants in BCT preferences for physical activity by users’ intention to change behavior and behavioral adoption

|  | **Physical activity** | | | **LPA** | **MVPA** |
| --- | --- | --- | --- | --- | --- |
| Behavior change technique | **Pre-intention (n=8)**  **M**±**SD** | **Post-intention (n=77)**  **M**±**SD** | **F ; *P*** | **Behavior**  **r ; *P***  **(n=78)** | **Behavior**  **r ; *P***  **(n=78)** |
| BCT1: Info behavior-health outcome | M=4.38  ± 0.52 | M=4.53  ± 0.60 | 0.52;  *.48* | -0.05;  *.67* | -0.00;  *.99* |
| BCT2: Self-monitoring of behavior | M=4.13  ± 0.64 | M=4.61  ± 0.59 | **4.86;**  ***.03*** | -0.04;  *.72* | 0.04;  *.72* |
| BCT3: Feedback on how well I do with PA | M=4.25  ± 0.46 | M=4.36  ± 0.76 | 0.17;  *.68* | 0.03;  *.82* | -0.07;  *.52* |
| BCT11: Getting insight in differences between what I do and what is needed to achieve the desired outcome | M=4.00  ± 1.07 | M=4.36  ± 0.71 | 1.74;  *.19* | 0.10;  *.37* | -0.01;  *.96* |
| BCT18: Getting tips tailored to my profile in relation to PA | M=4.00  ± 0.54 | M=4.13  ± 0.80 | 0.20;  *.66* | 0.05;  *.64* | -0.06;  *.63* |
| BCT10: Regular feedback on how my PA contributes to the desired outcome | M=4.00  ± 1.07 | M=4.16  ± 0.86 | 0.23;  *.63* | 0.05;  *.68* | -0.02;  *.89* |
| BCT4: Instructions how to improve PA | M=3.88  ± 0.84 | M=4.23  ± 0.84 | 1.32;  *.25* | -0.11;  *.34* | -0.04;  *.76* |
| BCT6: Adjusting personal goals | M=3.63  ± 1.19 | M=4.04  ± 0.98 | 1.25;  *.27* | 0.08;  *.51* | 0.03;  *.80* |
| BCT5: Setting personal goals | M=3.88  ± 0.84 | M=4.08  ± 1.00 | 0.31;  *.58* | -0.03;  *.78* | -0.01;  *.95* |
| BCT8: Setting a personally desired outcome | M=3.63  ± 1.30 | M=4.09  ± 1.02 | 1.45;  *.23* | -0.06;  *.61* | 0.04;  *.71* |
| BCT13: Identifying barriers for PA | M=3.75  ± 1.28 | M=3.92  ± 0.96 | 0.22;  *.64* | -0.00;  *.99* | -0.01;  *.94* |
| BCT9: Adjusting my personally desired outcome | M=3.63  ± 1.41 | M=3.92  ± 0.98 | 0.61;  *.44* | -0.00;  *.98* | 0.04;  *.75* |
| BCT7: Gradually building up to more difficult goals | M=3.63  ± 1.19 | M=3.86  ± 1.07 | 0.33;  *.57* | -0.07;  *.56* | 0.09;  *.43* |
| BCT17: Getting time management tips that help me improve my PA | M=3.50  ± 1.20 | M=3.77  ± 1.01 | 0.49;  *.49* | 0.19;  *.09* | -0.05;  *.64* |
| BCT20: Getting a reminder when it is time to do something about my PA | M=3.38  ± 0.92 | M=3.69  ± 1.15 | 0.56;  *.46* | 0.06;  *.62* | -0.07;  *.55* |
| BCT21: That the app provides encouragement and helps to keep it up | M=3.50  ± 1.20 | M=3.75  ± 1.04 | 0.42;  *.52* | 0.10;  *.37* | -0.11;  *.36* |
| BCT12: Creating an action plan for PA | M=3.75  ± 1.28 | M=3.77  ± 1.08 | 0.00;  *.97* | -0.07;  *.56* | -0.11;  *.35* |
| BCT15: To compare myself with others with a similar profile of PA | M=3.50  ± 1.30 | M=3.25  ± 1.23 | 0.31;  *.58* | 0.06;  *.61* | 0.00;  *.97* |
| BCT22: Getting a reward, incentive or appreciation when I make progress in relation to PA | M=3.13  ± 1.13 | M=3.29  ± 1.22 | 0.13;  *.72* | 0.08;  *.50* | -0.18;  *.12* |
| BCT19: Getting video’s that show me how to improve my PA | M=3.00  ± 1.20 | M=3.17  ± 1.19 | 0.15;  *.70* | -0.04;  *.76* | **-0.33;**  ***.004*** |
| BCT14: Getting social support to improve my PA | M=3.50  ± 1.07 | M=3.03  ± 1.14 | 1.28;  *.26* | -0.02;  *.90* | -0.21;  *.07* |
| BCT16: That I can be an example to others, inspire or motivate them for PA | M=2.75  ± 0.89 | M=2.74  ± 1.22 | 0.00;  *.98* | -0.10;  *.41* | -0.02;  *.90* |
| EF7: Instructions from virtual coach | M=3.75  ± 0.71 | M=3.11  ± 1.27 | *NH* | 0.09;  *.42* | **-0.43;**  ***<.001*** |
| EF2: Competition with others | M=2.25  ± 0.89 | M=2.61  ± 1.21 | *NH* | -0.15;  *.20* | 0.06;  *.61* |
| EF8: Asking questions via chat | M=2.25  ± 0.89 | M=2.54  ± 1.16 | *NH* | 0.07;  *.53* | -0.20;  *.07* |
| EF1: Gamification | M=2.38  ± 0.92 | M=2.57  ± 1.01 | 0.26;  *.61* | 0.11;  *.35* | -0.17;  *.14* |
| EF3: Narrative | M=1.75  ± 0.71 | M=1.66  ± 0.87 | 0.08;  *.77* | 0.05;  *.66* | -0.11;  *.35* |
| EF4: Character in a narrative | M=1.63  ± 0.52 | M=1.66  ± 0.84 | 0.01;  *.91* | 0.12;  *.29* | -0.19;  *.09* |
| EF5: Support by celebrities | 1.63  0.52 | M=1.43  ± 0.70 | 0.56;  *.46* | 0.16;  *.15* | -0.21;  *.07* |
| EF6: Connection to social media | M=2.00  ± 1.07 | M=1.63  ± 0.73 | 1.69;  *.20* | 0.04;  *.71* | -0.06;  *.63* |

NH: No homogeneity of variances

(df): 1,83

BCT: behavior change technique; EF: engagement feature
