# Supplementary material for: Adults’ Preferences for Behavior Change Techniques and Engagement Features in a Mobile App to Promote 24-Hour Movement Behaviors: Cross-Sectional Survey Study
Source: JMIR Mhealth Uhealth. 2019 Dec 20;7(12):e15707. doi: 10.2196/15707 (PMC6942183; doi:10.2196/15707)
Supplement: Multimedia Appendix 5 [file mhealth_v7i12e15707_app5.docx]

**Multimedia Appendix 5.** Differences between participants in BCT preferences for sleep by users’ intention to change behavior and behavioral adoption

|  | **Pre-intention (n=35)** | **Post-intention (n=48)** | **F ; *P*** | **Behavior**  **r *; P***  **(n=78)** |
| --- | --- | --- | --- | --- |
| BCT1: Info behavior-health outcome | M= 4.49  ± 0.70 | M= 4.44  ± 0.68 | 0.10;  *.75* | **-0.23;**  ***.048*** |
| BCT2: Self-monitoring of behavior | M=4.54  ± 0.66 | M=4.46  ± 0.71 | 0.30;  *.58* | **-0.25;**  ***.03*** |
| BCT3: Feedback on how well I do with sleep | M=4.43  ± 0.66 | M=4.17  ± 0.69 | 3.02;  *.09* | **-0.23;**  ***.04*** |
| BCT11: Getting insight in differences between what I do and what is needed to achieve the desired outcome | M=4.37  ± 0.60 | M=4.13  ± 0.82 | 2.29;  *.13* | -0.10;  *.40* |
| BCT18: Getting tips tailored to my profile in relation to sleep | M=4.14  ± 0.77 | M=4.00  ± 0.72 | 0.76;  *.39* | 0.01;  *.92* |
| BCT10: Regular feedback on how my sleep contributes to the desired outcome | M=4.26  ± 0.78 | M=3.98  ± 0.79 | 2.55;  *.11* | -0.06;  *.59* |
| BCT4: Instructions how to improve sleep | M=4.23  ± 0.77 | M=4.13  ± 0.89 | 0.31;  *.58* | -0.04;  *.73* |
| BCT6: Adjusting personal goals | M=3.89  ± 0.83 | M=3.71  ± 1.07 | *NH* | -0.05;  *.67* |
| BCT5: Setting personal goals | M=3.91  ± 0.95 | M=3.71  ± 0.90 | 1.01;  *.32* | -0.08;  *.47* |
| BCT8: Setting a personally desired outcome | M=3.83  ± 1.10 | M=3.85  ± 1.01 | 0.01;  *.91* | -0.06;  *.59* |
| BCT13: Identifying barriers for sleep | M=4.06  ± 0.84 | M=3.79  ± 0.97 | 1.70;  *.20* | -0.03;  *.78* |
| BCT9: Adjusting my personally desired outcome | M=3.80  ± 0.87 | M=3.58  ± 0.99 | 1.08;  *.30* | -0.06;  *.58* |
| BCT7: Gradually building up to more difficult goals | M=3.60  ± 0.81 | M=3.52  ± 1.03 | *NH* | -0.14;  *.24* |
| BCT17: Getting time management tips that help me improve my sleep | M=3.80  ± 0.99 | M=3.60  ± 1.01 | 0.78;  *.38* | 0.05;  *.64* |
| BCT20: Getting a reminder when it is time to do something about my sleep | M=3.66  ± 1.11 | M=3.19  ± 1.16 | 3.44;  *.07* | -0.03;  *.78* |
| BCT21: That the app provides encouragement and helps to keep it up | M=3.31  ± 1.02 | M=3.42  ± 0.92 | 0.23;  *.63* | -0.03;  *.78* |
| BCT12: Creating an action plan for sleep | M=3.57  ± 1.09 | M=3.52  ± 0.97 | 0.05;  *.82* | -0.01;  *.91* |
| BCT15: To compare myself with others with a similar profile of sleep | M=3.37  ± 1.22 | M=3.08  ± 1.25 | 1.10;  *.30* | -0.03;  *.78* |
| BCT22: Getting a reward, incentive or appreciation when I make progress in relation to sleep | M=3.29  ± 1.07 | M=2.92  ± 1.07 | 2.41;  *.13* | 0.02;  *.87* |
| BCT19: Getting video’s that show me how to improve my sleep | M=2.91  ± 1.27 | M=2.92  ± 1.24 | 0.00;  *.99* | 0.19;  *.09* |
| BCT14: Getting social support to improve my sleep | M=2.66  ± 1.19 | M=2.79  ± 1.15 | 0.27;  *.61* | **0.23;**  ***.048*** |
| BCT16: That I can be an example to others, inspire or motivate them for sleep | M=2.63  ± 1.22 | M=2.65  ± 1.04 | 0.01;  *.95* | 0.01;  *.93* |
| EF7: Instructions from virtual coach | M=3.11  ± 1.18 | M=3.19  ± 1.30 | 0.07;  *.79* | 0.17;  *.15* |
| EF2: Competition with others | M=2.77  ± 1.24 | M=2.44  ± 1.15 | 1.60;  *.21* | -0.05;  *.65* |
| EF8: Asking questions via chat | M=2.63  ± 1.00 | M=2.44  ± 1.24 | 0.57;  *.46* | 0.16;  *.16* |
| EF1: Gamification | M=2.66  ± 1.03 | M=2.44  ± 0.97 | 0.99;  *.32* | 0.17;  *.14* |
| EF3: Narrative | M=1.63  ± 0.88 | M=1.65  ± 0.79 | 0.01;  *.93* | 0.19;  *.09* |
| EF4: Character in a narrative | M=1.71  ± 0.96 | M=1.60  ± 0.71 | 0.36;  *.55* | 0.19;  *.10* |
| EF5: Support by celebrities | M=1.40  ± 0.60 | M=1.48  ± 0.74 | 0.27;  *.61* | 0.15;  *.21* |
| EF6: Connection to social media | M=1.66  ± 0.80 | M=1.67  ± 0.75 | 0.00;  *.96* | 0.15;  *.18* |

NH: No homogeneity of variances

(df): 1,81

BCT: behavior change technique; EF: engagement feature
