# Supplementary material for: Adults’ Preferences for Behavior Change Techniques and Engagement Features in a Mobile App to Promote 24-Hour Movement Behaviors: Cross-Sectional Survey Study
Source: JMIR Mhealth Uhealth. 2019 Dec 20;7(12):e15707. doi: 10.2196/15707 (PMC6942183; doi:10.2196/15707)
Supplement: Multimedia Appendix 6 [file mhealth_v7i12e15707_app6.docx]

**Multimedia Appendix 6.** Differences between participants in BCT preferences for sedentary behavior by users’ intention to change behavior and behavioral adoption

| Behavior Change Technique | **Pre-intention (n=44)** | **Post-intention (n=41)** | **F ; *P*** | **Behavior**  **r *; P***  **(n=78)** |
| --- | --- | --- | --- | --- |
| BCT1: Info behavior-health outcome | M=4.09  ±0.94 | M=4.29  ± 0.72 | 1.23;  *.27* | 0.01;  *.94* |
| BCT2: Self-monitoring of behavior | M=4.11  ± 0.95 | M=4.34  ± 0.79 | 1.44;  *.23* | 0.09;  *.43* |
| BCT3: Feedback on how well I do with SB | M=3.93  ± 1.02 | M=3.98  ± 0.96 | 0.04;  *.84* | 0.10;  *.38* |
| BCT11: Getting insight in differences between what I do and what is needed to achieve the desired outcome | M=3.95  ± 0.91 | M=4.07  ± 0.93 | 0.35;  *.56* | 0.11;  *.33* |
| BCT18: Getting tips tailored to my profile in relation to SB | M=3.95  ± 0.83 | M=3.88  ± 0.98 | 0.15;  *.70* | 0.04;  *.72* |
| BCT10: Regular feedback on how my SB contributes to the desired outcome | M=3.95  ± 0.91 | M=3.90  ± 0.97 | 0.07;  *.80* | -0.02;  *.89* |
| BCT4: Instructions how to improve SB | M=3.84  ± 1.03 | M=3.95  ± 1.02 | 0.24;  *.62* | 0.11  *.33* |
| BCT6: Adjusting personal goals | M=3.64  ± 1.04 | M=3.73  ± 1.12 | 0.17;  *.68* | -0.06;  *.60* |
| BCT5: Setting personal goals | M=3.59  ± 1.09 | M=3.73  ± 1.07 | 0.36;  *.55* | 0.13;  *.24* |
| BCT8: Setting a personally desired outcome | M=3.68  ± 1.03 | M=3.71  ± 1.17 | 0.01;  *.92* | 0.04;  *.74* |
| BCT13: Identifying barriers for SB | M=3.66  ± 0.89 | M=3.76  ± 1.04 | 0.21;  *.65* | -0.03  *.83* |
| BCT9: Adjusting my personally desired outcome | M=3.61  ± 1.04 | M=3.61  ± 1.05 | 0.00;  *.99* | -0.02;  *.85* |
| BCT7: Gradually building up to more difficult goals | M=3.41  ± 1.19 | M=3.61  ± 0.97 | 0.72;  *.40* | -0.06;  *.63* |
| BCT17: Getting time management tips that help me improve my SB | M=3.52  ± 1.07 | M=3.59  ± 1.10 | 0.07;  *.79* | -0.04;  *.74* |
| BCT20: Getting a reminder when it is time to do something about my SB | M=3.43  ± 1.17 | M=3.27  ± 1.32 | 0.37;  *.55* | 0.12;  *.30* |
| BCT21: That the app provides encouragement and helps to keep it up | M=3.34  ± 1.16 | M=3.44  ± 1.14 | 0.15;  *.70* | 0.01;  *.92* |
| BCT12: Creating an action plan for SB | M=3.43  ± 1.07 | M=3.54  ± 1.10 | 0.20;  *.66* | 0.10;  *.41* |
| BCT15: To compare myself with others with a similar profile of SB | M=2.95  ± 1.20 | M=3.22  ± 1.22 | 1.02;  *.32* | -0.07;  *.54* |
| BCT22: Getting a reward, incentive or appreciation when I make progress in relation to SB | M=2.82  ± 1.11 | M=3.24  ± 1.32 | 2.62;  *.11* | 0.04;  *.75* |
| BCT19: Getting video’s that show me how to improve my SB | M=3.00  ± 1.28 | M=2.93  ± 1.21 | 0.07;  *.79* | 0.04  *.75* |
| BCT14: Getting social support to improve my SB | M=2.73  ± 1.15 | M=2.93  ± 1.15 | 0.64;  *.43* | 0.02;  *.87* |
| BCT16: That I can be an example to others, inspire or motivate them for SB | M=2.45  ± 1.09 | M=2.80  ± 1.10 | 2.18;  *.14* | 0.08;  *.51* |
| EF7: Instructions from virtual coach | M=3.11  ± 1.28 | M=3.23  ± 1.21 | 0.17;  *.68* | -0.01;  *.93* |
| EF2: Competition with others | M=2.57  ± 1.04 | M=2.58  ± 1.34 | *NH* | 0.12;  *.30* |
| EF8: Asking questions via chat | M=2.59  ± 1.09 | M=2.43  ± 1.20 | 0.44;  *.51* | -0.07;  *.53* |
| EF1: Gamification | M=2.55  ± 1.00 | M=2.55  ± 1.01 | 0.00;  *.98* | -0.10;  *.40* |
| EF3: Narrative | M=1.82  ± 0.92 | M=1.50  ± 0.75 | 2.97;  *.09* | -0.07;  *.52* |
| EF4: Character in a narrative | M=1.75  ± 0.78 | M=1.55  ± 0.85 | 1.27;  *.26* | -0.09;  *.42* |
| EF5: Support by celebrities | M=1.55  ± 0.70 | M=1.35  ± 0.66 | 1.73;  *.19* | -0.06;  *.62* |
| EF6: Connection to social media | M=1.68  ± 0.77 | M=1.65  ± 0.77 | 0.04;  *.85* | -0.12;  *.30* |

NH: No homogeneity of variances

(df): 1, 83

BCT: behavior change technique; EF: engagement feature
